# Use of biochar and a post-coagulation effluent as an adsorbent of malachite green, beneficial bacteria carrier, and seedling substrate for plants belonging to the poaceae family

**DOI:** 10.1007/s13205-023-03766-x

**Published:** 2023-11-03

**Authors:** Christy A. Plaza-Rojas, Nelson A. Amaya-Orozco, Claudia M. Rivera-Hoyos, José S. Montaña-Lara, Adriana Páez-Morales, Juan Carlos Salcedo-Reyes, Laura C. Castillo-Carvajal, Wilmar Martínez-Urrútia, Lucía Ana Díaz-Ariza, Aura M. Pedroza-Rodríguez

**Affiliations:** 1 Laboratorio de Microbiología Ambiental y Suelos, Unidad de Investigaciones Agropecuarias (UNIDIA), Departamento de Microbiología, Facultad de Ciencias, Pontificia Universidad Javeriana, Carrera 7ma No 43-82, Edifício 50 Lab. 106, P.O. Box 110-23, Bogotá, DC Colombia; 2 Laboratorio de Biotecnología Molecular, Grupo de Biotecnología Ambiental e Industrial (GBAI), Departamento de Microbiología, Facultad de Ciencias, Pontificia Universidad Javeriana, P.O. Box 110-23, Bogotá, DC Colombia; 3 Laboratorio de Películas Delgadas y Nanofotónica, Grupo de Películas Delgadas y Nanofotónica, Departamento de Física, Facultad de Ciencias, Pontificia Universidad Javeriana, P.O. Box 110-23, Bogotá, DC Colombia; 4 Facultad de Ciencias de la Salud, Universidad Anáhuac, P.O. Box 01840, México, DF México; 5 Grupo de Diseño Avanzado, Fundación Universidad de América, P.O. Box 110-23, Bogotá, DC Colombia; 6 Laboratorio Asociaciones Suelo-Panta-Microorganismo, Grupo de Investigación en Agricultura Biológica, Departamento de Biología, Facultad de Ciencias, Pontificia Universidad Javeriana, P.O. Box 110-23, Bogotá, DC Colombia

**Keywords:** Fungal/bacterial consortium, *Chlorella* sp., Biogenic biomass, Lignocellulosic biomass, Co-pyrolysis, Bacteria carrier and seedling substrate

## Abstract

Wastewater treatment plants produce solid and semi-solid sludge, which treatment minimises secondary environmental pollution because of wastewater treatment and obtaining new bioproducts. For this reason, in this paper, the co-pyrolysis of biogenic biomasses recovered from a biological reactor with immobilised fungal and bacterial biomass and a tertiary reactor with *Chlorella* sp. used for dye-contaminated wastewater treatment was carried out. Biogenic biomasses mixed with pine bark allowed the production and characterisation of two types of biochar. The raw material and biochar were on the “in vitro” germination of *Lolium* sp. seeds, followed by adsorption studies for malachite green (MG) dye using the raw material and the biochar. Results showed that using 60 mg L^−1^ of a cationic coagulant at pH 6.5 allowed for the recovery of more than 90% of the microalgae after 50 min of processing. Two biochar resulted: BC_300_, at pH 5.08 ± 0.08 and BC_500_, at pH 6.78 ± 0.01. The raw material and both biochars were co-inoculated with growth-promoting bacteria; their viabilities ranged from 1.7 × 10^6^ ± 1.0 × 10^1^ to 7.5 × 10^8^ ± 6.0 × 10^2^ CFU g^−1^ for total heterotrophic, nitrogen-fixing and phosphate-solubilising bacteria. Re-use tests on *Lolium* sp. seed germination showed that with the post-coagulation effluent, the germination was 100%, while with the biochar, with and without beneficial bacteria, the germination was 98 and 99%, respectively. Finally, BC_500_ adsorbed the highest percentage of malachite green at pH 4.0, obtaining *q*ecal values of 0.5249 mg g^−1^ (*R*^2^: 0.9875) with the pseudo-second-order model.

**Supplementary Information:**

The online version contains supplementary material available at 10.1007/s13205-023-03766-x.

## Introduction

Industrial development and the increase in the world’s population have led to a decrease in renewable resources, overexploitation of fossil fuels, an increase in greenhouse gases and the generation of large quantities of solid and liquid waste, including domestic and non-domestic wastewater (Sabeen et al. 2018; Hernández-Sáenz et al. 2020). The non-domestic wastewater received at the treatment plants passes through a series of unitary processes and operations classified into primary, secondary and tertiary treatment (Puentes-Morales et al. 2020). In these sequential processes, the wastewater reaches the degree of purity required according to the national standards of each country for discharge into sewage systems or to reuse the effluent as irrigation water for gardens, green areas, sports fields and restoration of eroded soils (Truchado et al. 2021). Additionally, another practice that has gained relevance is the treated water utilisation in crop irrigation for food production as long as it meets physical, chemical and microbiological quality criteria (Almuktar et al. 2018; Pedroza-Camacho et al. 2018; Helmecke et al. 2020; Leonel and Tonetti 2021). These new reuse alternatives can be easily integrated into circular economy models and progressively decrease discharge into sewage systems (IWA 2018; Helmecke et al. 2020; Mainardis et al. 2022).

In association with wastewater treatment, primary, secondary and tertiary treatment, the generated sludge has a variable chemical composition, depending on the amount of inorganic and organic compounds it contains (Djandja et al. 2021). These sludges must be removed from the treated effluent by physical processes such as conventional primary, secondary, or tertiary sedimentation or chemically assisted sedimentation (coagulation-flocculation) (Singh and Patidar 2018; Xiao et al. 2022). The coagulation-flocculation process involves cationic and or anionic coagulants, organic polymers (gums, moringa, chitosan, alginate, among others), inorganic compounds (sulphates and chlorides) and bio-flocculation with exopolysaccharide-producing microorganisms (Singh and Patidar 2018; Ejimofor et al. 2021; Mora-León et al. 2022).

Depending on the type of non-domestic wastewater treated and the coagulants used to recover the sludge, these sludges have varying concentrations of carbonaceous and nitrogenous organic matter, nutrients such as nitrates and orthophosphates, microorganisms and chemical compounds associated with the coagulants used (Martín-Díaz et al. 2020; Gopinath et al. 2021; Jellali et al. 2021). These chemical and microbiological characteristics make sludge wastes unsuitable for direct use, being necessary to stabilise them to reduce the environmental impact on soils and surface waters (Jellali et al. 2021). Physical processes such as incineration (Hao et al. 2019), chemical treatments with powerful-oxidising compounds (Hu et al. 2020) and biological such as anaerobic digestion in reactors or open composting (Guo et al. 2020; Jadhav et al. 2021) are frequent to stabilise sludge (Gherghel et al. 2019).

On the other hand, some research assumed the production and characterisation of new materials from sewage treatment plant sludge (Soria-Verdugo et al. 2017; Gherghel et al. 2019; Ejimofor et al. 2021; Gopinath et al. 2021; Jellali et al. 2021; Xiao et al. 2022). Especially those sources of renewable energy that help capture gaseous emissions and reincorporate slow-release forms of carbon into the ecosystem to act as carbon reservoirs (Soria-Verdugo et al. 2017; Bolognesi et al. 2021). In this sense, the production of biochar using different types of sludge from treatment plants as raw materials is a promising alternative because the thermal conversion of only sludge (simple pyrolysis of biogenic biomass) or in a mixture with other agro-industrial by-products (co-pyrolysis) can be carried out (Fakayode et al. 2020). In such a way, it results in a biochar with a high degree of condensation, stability, and a higher number of functional groups, which, when used in soils, could actively participate in the adsorption of pollutant compounds and favour the retention of water, nutrients and microorganisms (Fakayode et al. 2020; Gopinath et al. 2021; Jellali et al. 2021).

One of the most commonly used agro-industrial wastes for the co-pyrolysis process with sludge is lignocellulosic biomasses (tree bark, leaves, sawdust and shavings) generated in forestry companies (Moreno-Bayona et al. 2019; Castillo-Toro et al. 2021; Céspedes-Bernal et al. 2021). These wastes provide lignin, a resistant aromatic polymer with high carbon/nitrogen ratios and low biodegradability (Yoo et al. 2020; He et al. 2021), that once thermally transformed, allows biochar with higher slow-release carbon content to be obtained compared to biochar obtained using only biogenic biomass from secondary or tertiary sludge (Chen et al. 2017; Chakraborty et al. 2021).

In sustainable agriculture, biochar obtained by co-pyrolysis is destinated to soil conditioner, organic fertiliser, planting, and germination substrate in nurseries (Céspedes-Bernal et al. 2021), improving the physical and chemical properties of the soil and promoting the biological activity of plants associated with systemic resistance to physical, chemical and biological factors (Arshad et al. 2021; Medeiros et al. 2021). Biochar also serves as a physical supporting material for beneficial microorganisms that promote plant growth and help decrease the excessive use of chemicals in the soil (Moreno-Bayona et al. 2019; Blanco-Vargas et al. 2022). At the environmental level, biochar serves as an adsorbent removing dyes, heavy metals, pesticides, hydrocarbons, and emerging pollutants, among others (Zeng et al. 2018; Castillo-Toro et al. 2021). These benefits are because biochar has functional groups such as hydroxyls, carboxyls, ethers, amides, amines, alkyls, alkynes and carbonyls, which interact with pollutants favouring their removal from wastewater or directly from soils (Castillo-Toro et al. 2021; Cheng et al. 2021).

Therefore, this work aimed to produce and characterise two types of biochar using the co-pyrolysis process of a waste mixture composed of fungal and bacterial biomass immobilised on lignocellulose meshes (*Furcraea* *andina*: Fique), coagulated algal sludge and pine bark. On the other hand, as reuse alternatives, the effect of uncoagulated, coagulated effluent, biochar and raw material on the germination of *Lolium* sp. seeds were assayed. Finally, measurement of the biochar adsorption capacity for Malachite Green dye removal (MG) occurred.

## Materials and methods

### Source of raw materials

By-products generated in a pilot plant during the treatment of non-domestic wastewater located at the Pontificia Universidad Javeriana Bogotá, D.C., Colombia, were used. The plant consisted of a grease trap, a biological reactor containing fungal and bacterial biomass immobilised on lignocellulose (*Furcraea* *andina*: Fique) meshes, a secondary settler and a tertiary reactor with *Chlorella* sp. The treatment plant operated for six continuous months, and the worn lignocellulose meshes were recovered and used as one of the raw materials for biochar production, as presented later. The tertiary effluent was the source to recover the algal biomass through the coagulation experiments becoming another raw material for transformation into biochar.

### Recovery of algal sludge

#### Selection of coagulants for algal sludge recovery

The evaluation involved five commercial coagulants: cationic coagulant based on polyacrylamide (Casa Químicos S.A.S.), guar gum (Proquímicas JG S.A.S.), xanthan gum (Disproalquímicos S.A.S), sodium alginate (Acros OrganicsTM) and FeCl_3_-6H_2_O (Merck^®^). The best coagulant selection was made by evaluating ascending concentrations of each product (50, 60, 70, 100, 150, 200, and 1500 mg L^−1^). The coagulation test occurred in 50 mL Schott flasks containing 20 mL of the microalgae suspension, and the doses of each coagulant were to be evaluated, with an initial pH of 6.5 ± 0.7 (pH of the tertiary effluent). After each coagulant addition, rapid mixing by stirring with a 5.0 cm long magnetic bar for 1 min at 200 ± 3 rpm allows homogenization. Subsequently, stirring continued but at a slower speed (slow mixing at 100 ± 2 rpm for 1 h); finally, sedimentation was for another hour. The tertiary effluent without coagulant was used as a control and kept under the same conditions. During the slow mixing and sedimentation time, the sampling was every 10 min up to 2 h to determine the percentage of biomass recovery as a function of time (Fig. S1 Supplementary material). The sample's absorbances at 680 nm in triplicate by using a Genesis 20 spectrophotometer were collected; distilled water was the assay blank, and calculated the percent of biomass recovery (BR) was by using Eq. 1 (Gorin et al. 2015).

1 $${\text{BR }}\left(\%\right)= \frac{{\text{abs blank}}-{\text{abs sample}}}{{\text{abs blank}}} \times 100,$$

where BR is the biomass recovery expressed as a percentage (%), abs blank is the absorbance at 680 nm of the tertiary effluent without coagulant, and abs sample is the absorbance at 680 nm of the sample after the coagulation-flocculation process.

The coagulant selection criterion was the concentration in mg L^−1^ at which the recovery rate was > 90%. To determine whether significant differences were between the coagulants at different concentrations, we compared the means in SAS^®^ 9.0 software for Windows. The coagulated algal sludge (CAS) was preserved at 4 °C to serve as raw material (RM) for biochar production.

### Characterisation of tertiary effluent before and after the coagulation process

Chemical characterisation of the tertiary effluent before and after the coagulation process allows for determining if they complied with the quality parameters for treated wastewater reuse according to resolution 1207 of 2014 (MADS 2014). Each sample was centrifuged for 15 min at 10,000*g* in an Eppendorf 5702 centrifuge. Subsequently, the recovered supernatant assays involved pH (OAKTON-pH 510/ion series Benchtop Meters^®^ electrode), electrical conductivity using an OAKTON conductivity meter, total solids (TS), (APHA 2017), total suspended solids (TSS), (APHA 2017), chemical oxygen demand (COD) by HACH^®^ method 8000 (HACH 2022a), total organic carbon percentage by ignition (TOC) by the thermochemical oxidation technique with ammonium persulphate HACH^®^ method 10129 (HACH 2022b), total nitrogen (TN) using the persulphate digestion technique HACH^®^ method 10,072 (HACH 2022c), ammonium concentration by ammonium salicylate and ammonium cyanurate technique, HACH^®^ method 8155 (HACH 2022d), the concentration of NO_3_^−^ by Cadmium reduction, HACH^®^ method 8039 (HACH 2022e), NO_2_^−^ concentration by ferrous sulphide method, HACH^®^ method 8153 (HACH 2022f), the concentration of sulphates by the barium sulphate method, HACH^®^ method 8051 (HACH 2022g), the concentration of sulphides following HACH^®^ method 8131 (HACH 2022h) and the orthophosphates concentration by the ascorbic acid method, HACH® method 8048 (HACH 2022i). Both effluents (uncoagulated and post-coagulated) were at 4 ± 1 °C maintained for “in vitro” seed germination assays of *Lolium* sp.

### Biochar production and characterisation

The raw materials (RM) used for the production of biochar were from three sources, lignocellulosic meshes (*Furcraea* *andina*: Fique) that had adsorbed fungi and bacteria, coming from a biological reactor with immobilised fungal and bacterial biomass discarded due to wear and clogging after 6 months of operation (Lignocellulosic meshes: RM_1_/LCM), (Pedroza and Puentes 2018). The second RM was the coagulated algal sludge from a tertiary reactor used for non-domestic wastewater treatment (RM_2_/CAS), and the third RM was pine bark (RM_3_/PB) obtained from the mechanical debarking process of pine trees (Moreno-Bayona et al. 2019; Castillo-Toro et al. 2021).

For the production of biochar at 300 and 500 °C, were employed 80 ± 2 g of shredded lignocellulose meshes (RM_1_/LCM), 230 ± 3 g of coagulated algal sludge (RM_2_/CAS) and 540 ± 4 g of pine bark (RM_3_/PB). The three materials were placed in an aluminium tray and mixed manually for 10 min. The raw materials mixture was dried in a HACEB® oven at 70 ± 3 °C for 12 h and referred to as a dry raw material mixture (DRMM). Subsequently, 80 g of DRMM were placed in 250 g aluminium trays and put inside a 3 M® brand anaerobiosis hood containing a 3M^®^ anaerogen sachet. The oxygen displacement process took 12 h at 19 ± 2 °C (Moreno-Bayona et al. 2019; Céspedes-Bernal et al. 2021). Then, aluminium trays were located in independent batches inside a 20 L Labtech™ muffle to perform the co-pyrolysis process for 1 h at 300 ± 3 and 500 ± 3 °C (Céspedes-Bernal et al. 2021). After the heat treatment, the biochar was preserved in anaerobic hoods to prevent them from acquiring moisture (Castillo-Toro et al. 2021). Each of the raw materials, the DRMM and the two types of biochar were for pH (ICONTEC 2011) and percentile content of moisture (ICONTEC 2011) and total organic carbon percentage by ignition (TOC) (Schumacher 2002; Lal and Das 2016; Moreno-Bayona et al. 2019; Schumacher 2002; Lal and Das 2016; Moreno-Bayona et al. 2019) assayed. As part of the proximate analysis, the percentage of volatile carbon (VC) (ASTM 2007), ash percentage (Ash) (ASTM 2007) and fixed carbon percentage (FC) (Moreno-Bayona et al. 2019) were determined. Finally, biochar yield calculation was with Eq. 2 (Yang et al. 2017); the stereoscopic observations were made at 40X using a Carl Zeiss/Stemi 305^®^ stereoscope.

2 $${\gamma }_{\mathrm{Biochar}}\left(\%\right)= \frac{{M}_{2}}{{M}_{1}}\times 100,$$

where: *Y*_Biochar_ is the biochar yield, *M*_2_ is the dry weight of the biochar at the end of the heat treatment, and *M*_1_ is the initial weight of the DRMM (lignocellulose meshes, algal sludge and pine bark, dried).

### Co-inoculation of biochar with beneficial bacteria

Cell suspension for co-inoculation was composed of *Pseudomonas* *fluorescens* (CMPUJ 376: phosphate solubilising and plant growth promoting bacterium), *Bacillus* *licheniformis* (CMPUJ 385: phosphate solubilising bacterium) and *Azotobacter* sp. (LMAS 1: non-associative nitrogen-fixing bacterium). Twenty-five mL of Brain Heart Infusion Broth (BHI), with 0.1 mL of a suspension of each bacterium (1.0 × 10^3^ ± 1.0 × 10^1^ CFU mL^−1^), in 100 mL Erlenmeyer flasks allowed performing the inoculum, which incubated for 12 h at 30 ± 2 °C and 120 rpm in a Shaker New Brunswick Innova^®^ 44. Subsequently, the inoculum was mixed in a 1:1:1 ratio in a 0.5 L Youtility™ flask to obtain the beneficial bacteria consortium with an initial concentration of 1.0 × 10^6^ ± 1.0 × 10^2^ CFU mL^−1^ (Blanco-Vargas et al. 2020, 2021).

One hundred g of DRMM, BC_300_, and BC_500_ were put in separate aluminium containers with 80 mL of the beneficial bacteria consortium each (saturation volume). The materials were mixed and homogenised with sterile spatulas until a wet paste (moisture: 80 ± 4%) and incubated at 30 ± 2 °C for 12 h in an incubator (Memmert®) as a secondary reactivation protocol for the beneficial bacteria (Díaz et al. 2014). The count of immobilised colony forming units per gram of solid material (CFU g^−1^) was by the decimal dilution and surface seeding technique on brain heart infusion (BHI) agar (total heterotrophic bacteria), NFB agar (nitrogen-fixing bacteria), SMRS1 agar with bromocresol purple (phosphate-solubilising bacteria) and Pikovskaya agar (phosphate-solubilising bacteria) done. Petri dishes were incubated at 30 ± 2 °C for 48 h for colony counting (Blanco-Vargas et al. 2021).

### Evaluation of tertiary effluent and biochar for *Lolium* sp. seed germination

Evaluation of whether the tertiary effluent (without coagulation and after coagulation) and biochar (DRMM, BC_300_ and BC_500_, co-inoculated and not co-inoculated with beneficial bacteria) could be promissory for “in vitro” germination of *Lolium* sp. seeds. For the tertiary effluent, in the base of 90 mm Petri dishes were independently placed Whatman® filter papers (90 mm diameter) impregnated with 5 mL of uncoagulated tertiary effluent or 100% (v/v) and post-co-inoculation effluent (100% v/v); assay performed in triplicate, (Blanco-Vargas et al. 2020).

Germination tests associated with the biochar and DRMM without co-inoculating or co-inoculated with the beneficial bacteria were carried out in Petri dishes of 90 mm in diameter, placing seven g of each material with a moisture of 80 ± 4% (the same material served as control but with sterile distilled water). Subsequently, 25 *Lolium* sp. seeds were in 5 rows placed, and the Petri dishes were kept at 18 ± 2 °C in the dark for five days. The germination percentage (%) calculation involved the previously described methodology at the test beginning and end (Pedroza-Rodríguez 2022).

### Evaluation of Biochar and DRMM as adsorbents

Adsorption kinetics were performed with Malachite Green (MG) dye using DRMM, BC_300_ and BC_500_ as adsorbents at pH 4.0, 6.0 and 7.0 ± 0.2. 100 mL Schott flasks containing 25 mL of a solution of the MG with a concentration of 5.0 mg L^−1^ adjusted to the required pH using HCl (0.1 N) or NaOH (0.1 N) (Castillo-Toro et al. 2021). Then, we added 0.5 g of each adsorbent, and the containers were sealed and kept in agitation at 120 r.p.m. and 19 ± 2 °C (performed in triplicate). From each replicates assay, samples were of 0.5 mL from the beginning, every 3 min until completing 20 min and then every 10 min to finish at 100 min. Absorbances at 618 nm of centrifuged samples serve for calculating the MG concentration (mg L^−1^). Subsequently, Eq. 3 allowed calculating the *q* value (amount of dye adsorbed in mg g^−1^ for each material).

3 $$q=\frac{V\left({C}_{0}-{C}_{f}\right)}{X},$$

where *q* is the mg of MG adsorbed per g of each adsorbent (DRMM, BC_300_ and BC_500_), (mg g^−1^), *V* is the volume of the solution (in L), *C*_*f*_ is the final concentration of MG (mg L^−1^), *C*_*o*_ is the initial concentration of MG (mg L^−1^), *X* are the g of each adsorbent. The value obtained corresponds to the average of three replicates (Céspedes-Bernal et al. 2021).

Subsequently, Pseudo-first-order and Pseudo-second-order models were applied to evaluate which described best the MG adsorption process. The calculated parameters were the maximum amount of adsorbed dye (mg g^−1^) of biochar (*q*_*e*_), Pseudo-first-order adsorption coefficient (*k*_1_) and pseudo-second-order adsorption coefficient (*k*_2_) (Morales-Álvarez et al. 2016; Castillo-Toro et al. 2021; Blanco-Vargas et al. 2022). Equations 4 and 5 describe the models:

4 $${\text{Pseudo-first-order model: }} \, \mathrm{ Ln }\left(qe-q\right)=\mathrm{ln} \, qe-kt,$$

where *qe* is the amount of dye adsorbed at equilibrium (mg g^−1^), *q* is the dye adsorbed per gram of adsorbent (mg g^−1^), *k* is the pseudo-first-order adsorption coefficient (min^−1^), *t* is time (min).

5 $${\text{Pseudo-second-order model: }}\, \frac{t}{q} = \frac{1}{{k}_{2}{qe}^{2}} + \frac{1}{qe} t,$$

where *qe* is the amount of dye adsorbed at equilibrium (mg g^−1^), *q* is the dye adsorbed per gram of adsorbent (mg g^−1^), *k*^2^ is the pseudo-second-order adsorption coefficient (g mg ^−1^ min^−1^), *t* is time (min).

## Results

### Recovery of algal sludge

#### Selection of coagulants for algal sludge recovery

**Fig. 1:**
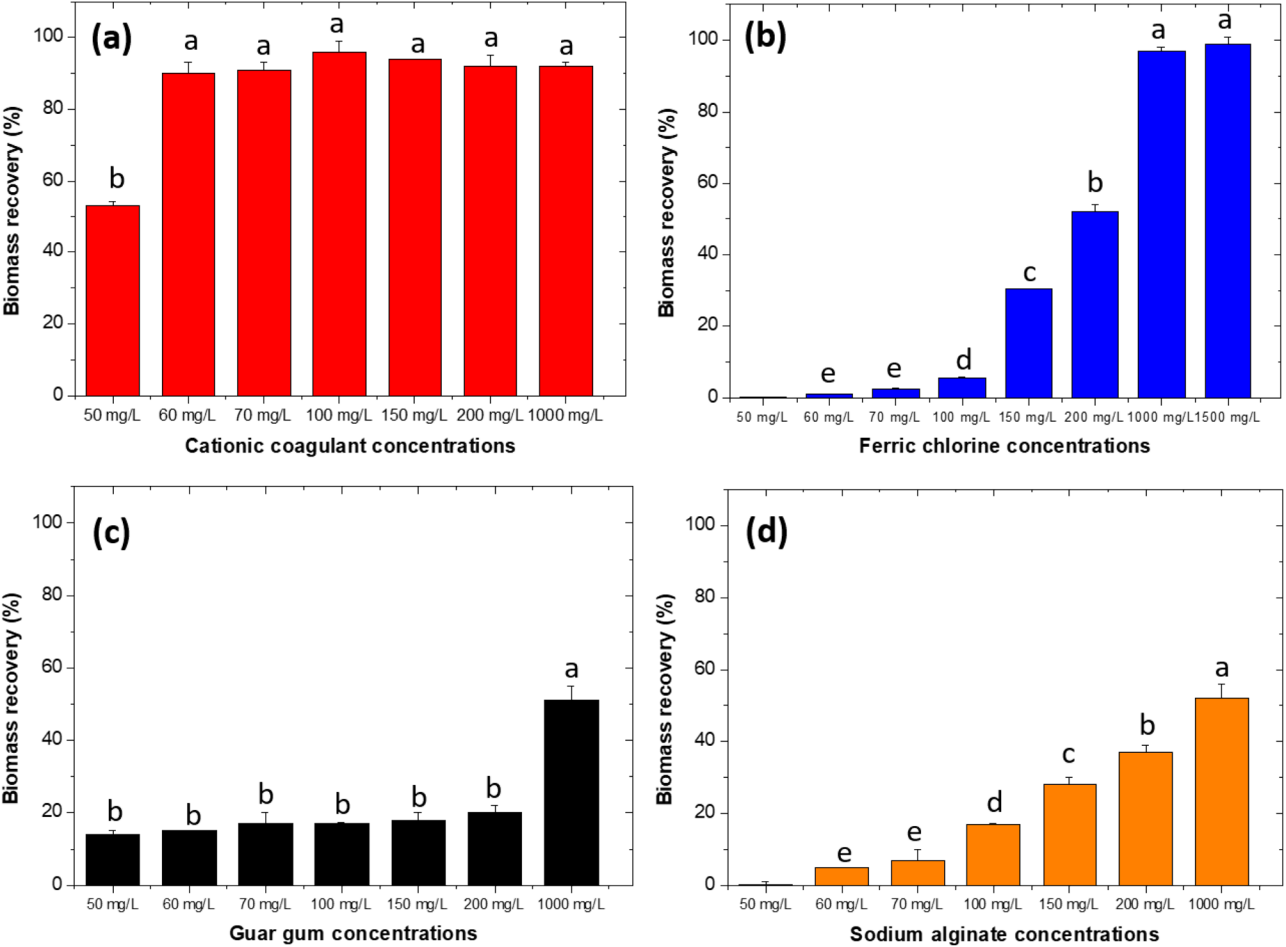
Biomass recovery RB (%) at increasing concentrations of the coagulants evaluated. **a** Cationic coagulant, **b** FeCl_3_-6H_2_O, **c** guar gum, **d** sodium alginate. Mean of three replicates ± SD. Lowercase letters represent significant differences between the doses of coagulants evaluated. The letter a identifies the best coagulants and their respective concentration in mg L^−1^

The uncoagulated tertiary effluent containing the suspended biomass of *Chlorella* sp. had a pH of 6.5 ± 0.8, TS 205 ± 10 mg L^−1^, TSS 199.7 ± 12 mg L^−1^ and SS 146.3 ± 9.2 mL L^−1^ (Table 1). Significant differences resulted between the coagulants (*p* < 0.0001) and the concentrations evaluated (*p* < 0.0001). The polyacrylamide-based cationic coagulant obtained the highest biomass recovery percentage (> 90%) from a concentration of 60 mg L^−1^ and after 50 min of agitation/sedimentation (Fig. 1a). At 120 min, the final percentage recovery was 97 ± 3% (Fig. S1a Supplementary material). Additionally, at the end of 120 min., the flocs formed were compact, large and did not disaggregate under strong shaking (Figs. S1b and S1c Supplementary material). From these flocs, samples were taken for optical microscopy (40 ×) and compared with the uncoagulated microalgae. In Fig. S2a Supplementary information, the microalgae were disaggregated and separated and compared with the aggregates in the presence of the cationic coagulant after the coagulation, the proportion of aggregates was superior when iron salt was present (Fig. S2b Supplementary material).

**Table 1:** Physical and chemical characterisation of tertiary effluent not coagulated and post-coagulated with 60 mg L^−1^ of cationic coagulant

Parameter	Units	Tertiary effluent not coagulated	Tertiary effluent post-coagulated	Resolution 1207 of 2014, Colombia Maximum permitted values	Title-40/chapter-I/subchapter-N EPA-USA
pH	-	6.5 ± 0.8	7.99 ± 0.02	6.0–9.0	6.0–9.0
TS	mg L^−1^	205 ± 10	15 ± 2	NS	NS
TSS	mg L^−1^	199.7 ± 12.1	13 ± 1	NS	45
SS	mL L^−1^	146.3 ± 9.2	1 ± 0	NS	1
Conductivity	µS cm^−1^	325 ± 21	138 ± 17	1500	NS
COD	mg L^−1^	205 ± 35.55	104.67 ± 3.67	NS	NS
BOD_5_	mg L^−1^	ND	ND	NS	30
TOC	mg L^−1^	29 ± 4.24	5.33 ± 1.05	NS	NS
NT	mg L^−1^	13 ± 2	7.0 ± 0.0	NS	NS
NO_3_^−^	mg L^−1^	2.33 ± 0.57	1.0 ± 0.0	5.0	NS
NO_2_^−^	mg L^−1^	2.67 ± 2.08	< 2.0 ± 0.0	NS	NS
NH_4_^+^	mg L^−1^	< 0.05 ± 0.00	< 0.05 ± 0.00	NS	7
SO_4_^2−^	mg L^−1^	56.67 ± 5.77	40 ± 2	500.0	NS
H_2_S	mg L^−1^	0.0206 ± 0.0013	0.00433 ± 0.0004	NS	NS
PO_4_^3−^	mg L^−1^	54.67 ± 1.52	47.33 ± 3.05	NS	NS

Resolution 1207 Ministry of Environment and Sustainable Development 2014. Environmental Protection Agency. Title−40/chapter-I/subchapter-N https://www.epa.gov/wqc/basic-information-water-quality-criteria and https://www.ecfr.gov/current/title-40/chapter-I/subchapter-N. 2023

*NS* not specified in the documents, ND *not* determined

For FeCl_3_-6H_2_O at pH 6.5 ± 0.2, only a recovery rate of 90 ± 4% was possible for 1000 and 1500 mg L^−1^ (Fig. 1b). The aggregates formed were laxer compared to those obtained with the cationic coagulant (Fig. S2c Supplementary material). For the three organic polymers, the highest percentages ranged between 50 ± 3 and 52 ± 4% for guar gum and sodium alginate at concentrations of 1000 mg L^−1^ (Fig. 1c, d). With xanthan gum, the highest efficiency was 22.5 ± 3% using 1000 mg L^−1^ (data not shown). From the experimental results, the cationic coagulant was better at a concentration of 60 mg L^−1^, pH 6.5 ± 0.2, and 2 h of coagulation process as these were the best conditions to recover the algal sludge for the production of the two types of biochar.

### Characterisation of tertiary effluent before and after the coagulation process

Table 1 shows the results of the tertiary effluent characterization without coagulation and the effluent after coagulation with the cationic coagulant at 60 mg L^−1^ at pH 6.5 ± 0.2. About the physical parameters, all solids were higher in the uncoagulated effluent with 205 ± 10 mg L^−1^, 199.7 ± 12.1 mg L^−1^ and 146.3 ± 9.2 mL L^−1^ for TS, TSS and SS. Solids in the post-coagulation effluent decreased by more than 90% (Table 1). Concerning the pH values, post-coagulation effluent ended with a pH of 7.99 ± 0.02 (1.49 units higher than the uncoagulated effluent 6.5 ± 0.04). A decrease in electrical conductivity (138 ± 17 µS cm^−1^) occurred in the post-coagulation effluent concerning the initial value (325 ± 21 µS cm^−1^) (Table 1). The coagulation-flocculation process also affected the decrease of COD and TOC, obtaining values of 104.67 ± 3.67 and 5.33 ± 1.05 mg L^−1^ (Table 1). Associated with the nitrogen cycle intermediates NT, NO_3_^−^, NO_2_^−^ and NH_4_^+^, the concentrations after the coagulation process were 7.0, 1.0, < 2.0 and < 0.05 mg L^−1^, for NT, NO_3_^−^, NO_2_^−^ and NH_4_^+^, respectively. The concentrations of SO_4_^−2^, H_2_S and PO_4_^−3^ slightly decreased if compared to the values obtained before the microalgae coagulation, reaching values of 40 ± 2, 0.00433 ± 0.0004 and 47.33 ± 3.05 mg L^−1^, respectively (Table 1). When analysing the results concerning resolution 1207 of 2014 (Colombian Ministry of Environment and Sustainable Development) that regulates the reuse of treated wastewater (MADS 2014), both uncoagulated and post-coagulated effluents were suitable for irrigation of pastures, fodder, green areas, and sports fields. However, the post-coagulated effluent had lower concentrations for the different parameters analysed, and the *Chlorella* sp. cells were more than 90%. Additionally, once analysed the results concerning the values reported in the Title-40/chapter-I/subchapter-N given by the US Environmental Protection Agency (EPA), it was proved that the post-coagulated effluent complies with the parameters pH, SS, TSS and NH_4_ concentration (Table 1).

### Biochar production and characterisation

The separated characterisation of lignocellulose meshes (RM_1_/LCM), coagulated algal sludge (RM_2_/CAS), pine bark (RM_3_/PB) and the dry mixture of them (DRMM) showed that pH values ranged from 7.1 ± 0.01 to 5.5 ± 0.07 for all raw materials (Table 2). The highest moisture content occurred in RM_2_/CAS (96.9 ± 0.59%), followed by RM_1_/LCM, RM_3_/PB and the mixture of them (DRMM), with values ranging between 9.04 ± 0.29, 7.43 ± 0.57 and 7.16 ± 1.04, respectively (Table 2). The raw materials containing lignocellulose had the highest TOC percentages with values of 48.7 ± 0.67, 41.7 ± 0.77 and 25.9 ± 0.08%, for RM_3_/PB, DRMM and RM_1_/LCM, respectively. On the contrary, the TOC percentage for the tertiary sludge of *Chlorella* sp. (RM_2_/CAS) was the lowest (2.72 ± 0.52%), which could be related to the low C:N ratio of this type of sludge (Table 2).

**Table 2:** Proximate analyzed for raw materials and biochar produced at 300 °C and 500 °C

Parameter	Units	Lignocelullosic mesh RM_1_/LCM	Coagulated algal sludge RM_2_/CAS	Pine bark RM_3_/PB	Mix of dry raw materials (DRMM)	BC_300_	BC_500_
pH	–	6.7 ± 0.11	7.1 ± 0.01	5.5 ± 0.07	6.4 ± 0.1	5.08 ± 0.08	6.78 ± 0.01
Moisture	(%)	9.04 ± 0.29	96.9 ± 0.59	7.43 ± 0.57	7.16 ± 1.04	3.7 ± 0.81	3.0 ± 0.02
TOC	(%)	25.9 ± 0.08	2.72 ± 0.52	48.7 ± 0.67	41.7 ± 0.77	27.6 ± 0.68	31.4 ± 1.23
VC	(%)	98.1 ± 0.28	92.7 ± 0.66	84.9 ± 1.25	65.4 ± 2.07	53.6 ± 0.18	51.2 ± 2.2
Ash	(%)	0.8 ± 0.08	5.26 ± 0.88	11.1 ± 0.98	10.1 ± 2.08	30.6 ± 2.4	38.4 ± 2.3
FC	(%)	1.1 ± 0.02	2.04 ± 0.81	4.0 ± 0.23	24.5 ± 0.44	15.8 ± 1.2	10.4 ± 1.1
Y_biochar_	(%)	UP	UP	UP	UP	52.6 ± 3.5	41.3 ± 2.4
Co-inoculation of the biochar with the beneficial bacteria							
Total bacteria in BHI agar	CFU g^−1^	UP	UP	UP	2.3 × 10^2^	7.5 × 10^8^	7.3 × 10^8^
PSB in SMRS1 agar	CFU g^−1^	UP	UP	UP	2.1 × 10^2^	2.5 × 10^8^	3.2 × 10^8^
PSB in Pikovskaya agar	CFU g^−1^	UP	UP	UP	3.3 × 10^2^	2.1 × 10^8^	2.2 × 10^8^
BFN in NFB agar	CFU g^−1^	ND	ND	ND	1.7 × 10^1^	1.7 × 10^6^	3.2 × 10^6^

UP: Undetermined parameter

The VC percentages for the raw materials ranged from 98.1 ± 0.28 to 65.4 ± 2.07% (Table 2). The ash content varied for the different types of raw materials, with the lowest values for lignocellulose (RM_1_/LCM) and *Chlorella* sp. tertiary sludge (RM_2_/CAS), with 0.8 ± 0.08 and 5.26 ± 0.88%, respectively. For pine bark (RM_3_/PB) and the mixture of dry raw materials (DRMM), the values were quite similar (11.1 ± 0.98 and 10.1 ± 2.08%). Finally, the percentage of FC ranged between (1.1 ± 0.02 and 24.5 ± 0.44%) for the four raw materials (Table 2).

When the biogenic and lignocellulosic biomasses were co-pyrolysed at 300 and 500 °C for 1 h, the biochars obtained showed different characteristics associated with the two temperatures evaluated. The pH for BC_300_ was lower (5.08 ± 0.08) than for BC_500_ (6.78 ± 0.01) (Table 2). The moisture percentage was lower for BC_500_ (3.0 ± 0.02%) than for BC_300_ (3.7 ± 0.81%). The TOC percentages were 27.6 ± 0.68 and 31.4 ± 1.23%, for BC_300_ and BC_500_, respectively. The VC and FC percentages decreased in the two types of biochar when compared to the dry raw material mixture, obtaining values of 53.6 ± 0.18, 15.8 ± 1.2, 51.2 ± 2.2 and 10.4 ± 1.1%, for VC and FC of BC_300_ and BC_500_, respectively (Table 2). On the contrary, the ash percentage increased in both types of biochar (30.6 ± 2.4 and 38.4 ± 2.3%, for BC_300_ and BC_500_) for the raw material (10.1 ± 2.08%). The biochar yield was higher in BC_300_ (52.6 ± 3.5%) than in BC_500_ (41.3 ± 2.4%), suggesting that at a lower temperature, less carbon is lost in the volatile fraction and concentrated in highly condensed carbon (Table 2).

**Fig. 2:**
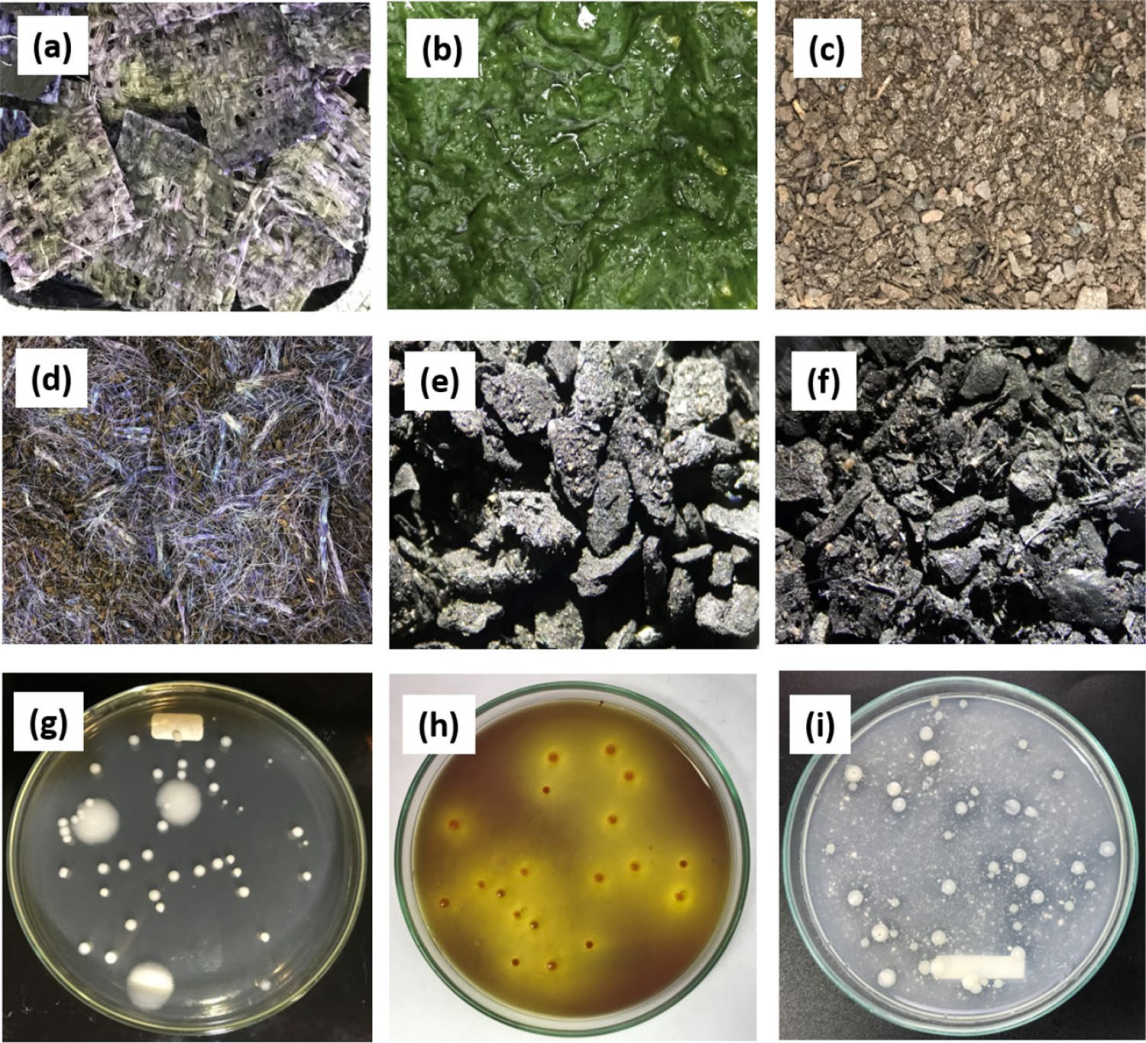
Macroscopic characteristics of raw materials, biochar and co-inoculated beneficial microorganisms. **a** RM_1_/LCM. **b** RM_2_/CAS. **c** RM_3_/PB. **d** DRMM. **e** BC_300_. **f** BC_500_. **g** Heterotrophic bacteria on BHI agar. **h** Phosphate solubilising bacteria on SMRS1 agar. **i** Phosphate solubilising bacteria on Pikovskaya agar

Figure 2a–c show the macroscopic appearance of the raw materials separately, with RM_1_/LCM in Fig. 2a, RM_2_/CAS in Fig. 2 and RM_3_/PB in Fig. 2c. Figure 2d showing the mixture of dry raw materials (DRMM) before the co-pyrolysis process and Fig. 2e, f show the macroscopic appearance of the biochar produced at 300 and 500 °C, respectively. Figure 2f shows that at higher temperatures (500 °C), it generates particle sizes lower than those from biochar at 300 °C (Fig. 2e).

### Co-inoculation of biochar with beneficial bacteria

The results of co-inoculation with the beneficial bacteria expressed in CFU g^−1^ are in Table 2. The initial counts in the dry raw material mixture (DRMM) were 2.3 × 10^2^ CFU g^−1^, 2.1 × 10^2^ CFU g^−1^, 3.3 × 10^2^ CFU g^−1^ and 1.7 × 10^1^ CFU g^−1^ for total bacteria, phosphate-solubilising bacteria on SMRS1 agar, Pikovskaya agar and nitrogen-fixing bacteria on NFB agar. The counts on the two types of biochar were higher and ranged from 1.7 × 10^6^ to 7.5 × 10^8^ CFU g^−1^ for the bacteria counted (Table 2). These results suggest that the dried raw materials (DRMM) may have adsorbed dyes or intermediates, which affected the viability of the beneficial bacteria compared to the two types of biochar because a decrease ranging from five to six log units was observed (Table 2).

Figure 2g shows the colonies of total heterotrophic bacteria, differentiating between Gram-positive bacilli (large colonies) and Gram-negative bacilli (small ones). Figure 2h, i show PSB colonies on SMRS1 agar (with solubilisation and acidification halos) and Pikovskaya agar (with solubilisation halos). These colonies recovered from both biochar types demonstrate that the co-inoculation protocol allowed immobilisation of the bacteria and no decrease in morphotypes or semi-quantitative biological activity. Therefore, the co-inoculated biochar proved to be ready for seed germination tests.

### Evaluation of tertiary effluent and biochar for *Lolium* sp. seed germination

The two tertiary effluents (uncoagulated effluent and post-coagulated with the cationic coagulant at 60 mg L^−1^) favoured the germination of *Lolium* sp. seeds, obtaining percentages of 94 ± 1.7 and 93 ± 2.3%, for the uncoagulated and post-coagulated effluent, respectively; values significantly higher than the control with distilled water (90 ± 1.6%), (*p* < 0.0001), (Table 3). When using both biochars, the germination percentage was 99%, while by adding bacteria, it oscillated between 98 and 99%, a higher result than the soil control (90 ± 2.31%, *p* < 0.0001). On the other hand, an adverse effect on *Lolium* sp. seed germination occurs when using the mixture of dry raw materials (DRMM) with and without bacteria, obtaining percentages lower than 35% after five days of evaluation (20 ± 3 and 30 ± 4%, for DRMM without bacteria and with bacteria), (Table 3).

**Table 3:** Use results of effluent and solid materials on the germination of *Lolium* sp. seeds for 5 days at 19 °C

Evaluated samples	
Treatments and control	*Lolium* sp. seed germination (%)
Uncoagulated tertiary effluent	**94 ± 1.7**^**a**^
Post-coagulation effluent	**93 ± 2.3**^**a**^
Distilled water control	90 ± 1.6
DRMM and biochar	
Bacteria-free DRMM	20 ± 3^d^
DRMM with bacteria	30 ± 4^c^
Bacteria-free BC_300_	**99 ± 3**^**a**^
Bacteria-free BC_500_	**99 ± 2**^**a**^
BC_300_ with bacteria	**99 ± 3**^**a**^
BC_500_ with bacteria	98 ± 3.0^a,b^
Control with soil	90 ± 2.0

Average of three replicates. Lowercase letters signify significant differences between treatments concerning controls. The bold letter indicates the best treatments

### Evaluation of Biochar and DRMM as adsorbents

**Fig. 3:**
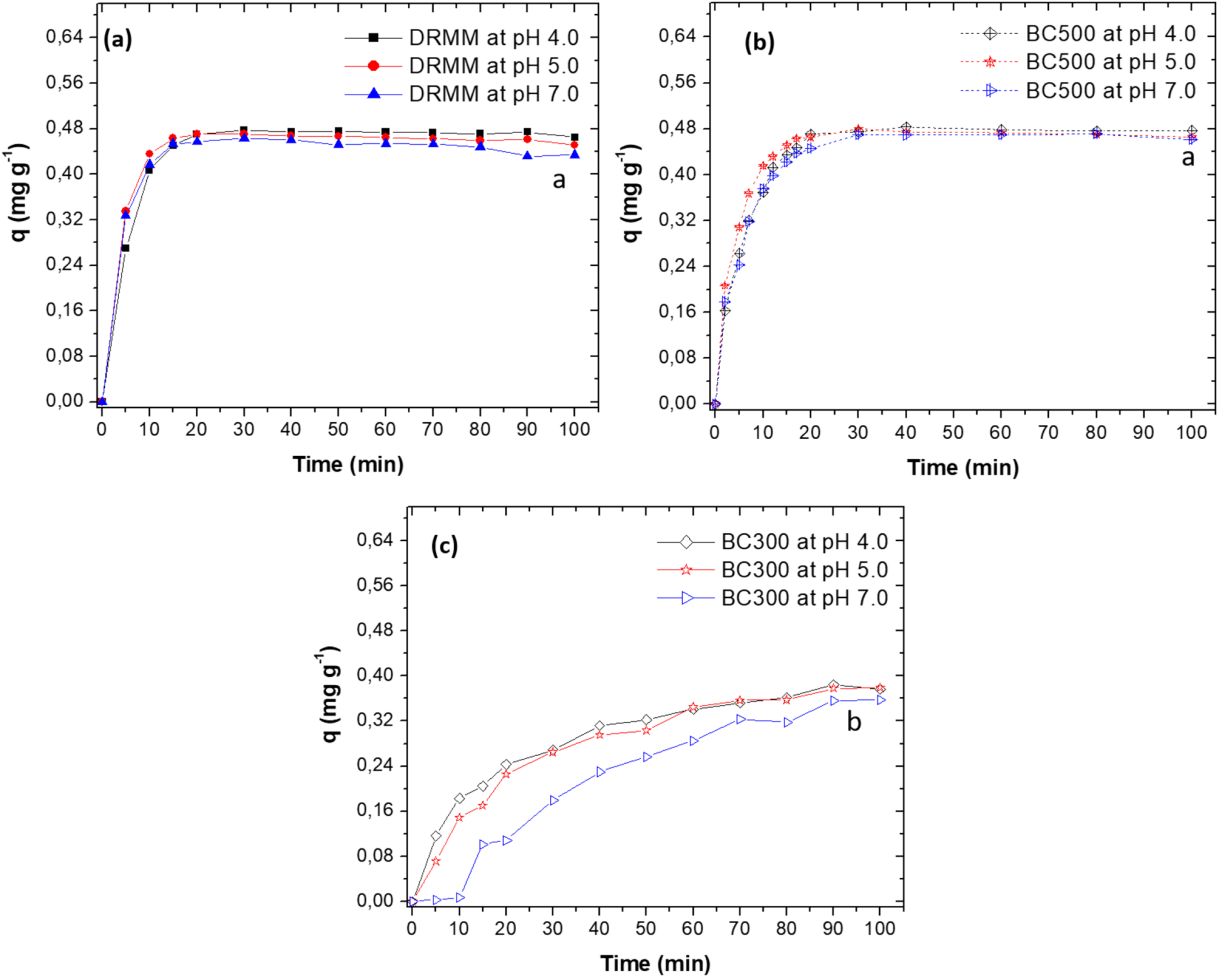
*q* value versus time at three pHs. Effect of three adsorbents on the MG adsorption. **a** DRMM. **b** BC_500_ and **c** BC_300_. Results presented correspond to the mean of three replicas. Lowercase letters represent significant differences between pH for each of the adsorbents tested. Regression data available in Table 4 and Table S1 for BC_300_ (Supplementary material)

DRMM and BC_500_ adsorbed the highest amount of the dye MG at the three pH values evaluated, reaching the adsorption/desorption equilibrium at 15 and 20 min of contact. Between 20 and 100 min, the experimental values of *qe* did not have a significant variation for DRMM and BC_500_, only in DRMM at pH 7.0 ± 0.2 between 80 and 100 min., a slight decrease was observed that could be related to dye desorption by saturation of active sites (Fig. 3a, b).

The adsorption process from MG to BC_300_ was slow, and the adsorbed amounts were also low, resulting in equilibrium reaching between 90 to 100 min of contact for all three pH values (Fig. 3c).

**Fig. 4:**
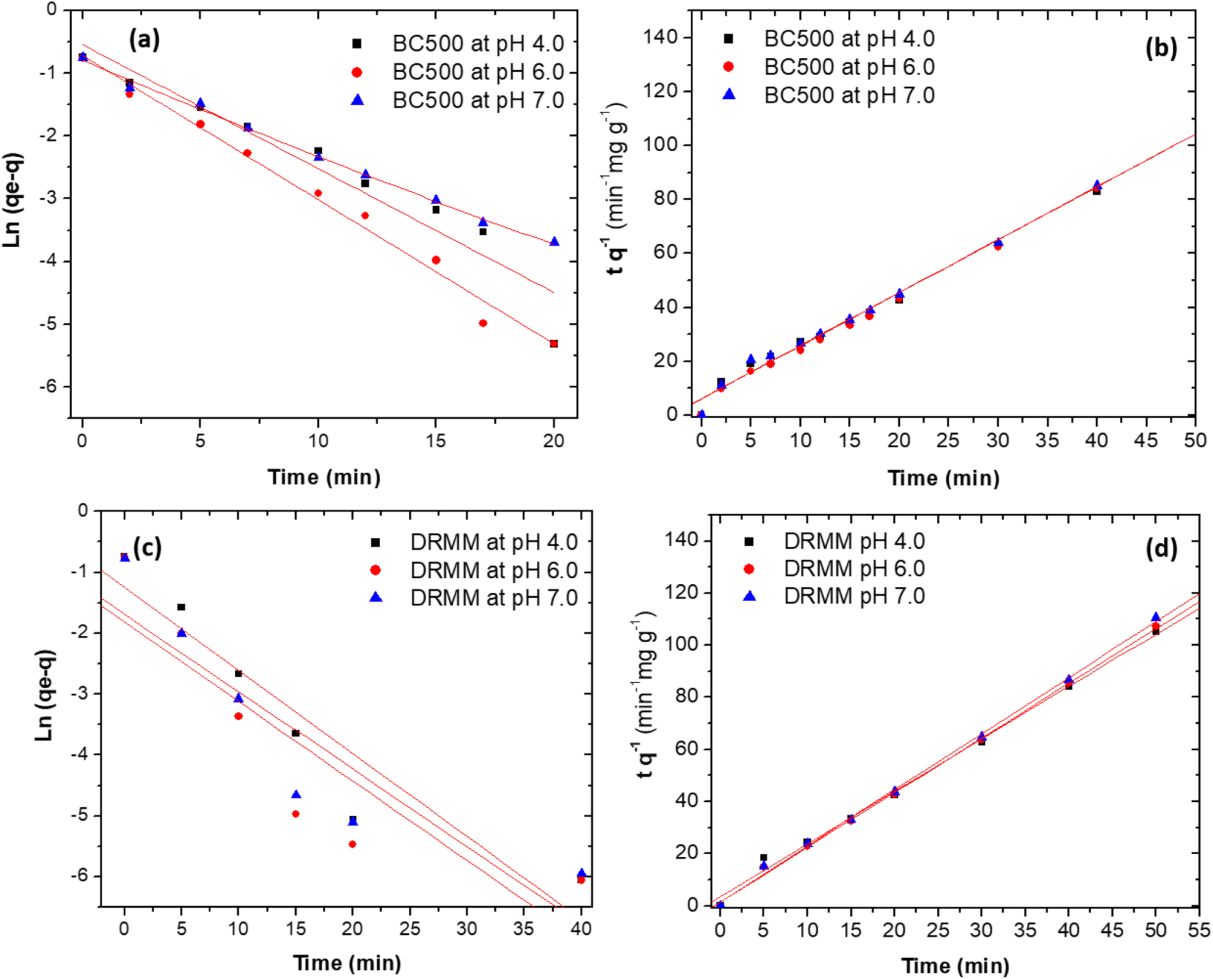
Kinect models and rate constants. Pseudo-first-order models for: **a** BC_500_, **b** pseudo-second-order models for BC_500_, **c** pseudo-first-order models for DRMM, **d** pseudo-second-order models for DRMM. At pH of 4.0, 6.0 and 7.0 ± 0.2

Concerning the pH values evaluated, no significant differences occurred between them for DRMM and BC_500_ (*p* > 0.0001) (Figs. 3a, 3b). However, at pH 4.0 ± 0.2, BC_500_ adsorbed more MG with *qe*_cal_ values of 0.5773 mg g^−1^ (*R*^2^ = 0.9302) and 0.5249 mg g^−1^ (*R*^2^ = 0.9875), when analysed the results of the Pseudo- first-order and Pseudo-second-order models, the adsorption constants were *k*_1_ = 0.1975 min^−1^ and *k*_2_ = 0.3020 g mg^−1^ min^−1^, respectively (Table 4, Fig. 4a, b).

**Table 4:** Results of kinetic study for the adsorption of MG onto BC_500_ and DRMM. Rate constants of pseudo-first-order and pseudo-second-order models at pH of 4.0, 6.0 and 7.0 ± 0.2

pH ± 0.2	Pseudo-first-order			Pseudo-second-order		
	*q*_*e*_ (mg g^−1^)	*K*_1_ (min^−1^)	*R*^2^	*q*_*e*_ (mg g^−1^)	*k*_2_ (g mg ^−1^ min^−1^)	*R*^2^
BC_500_						
4.0	0.5773	0.1975	0.9302	0.5249	0.3020	0.9875
6.0	0.4882	0.2296	0.9877	0.5029	0.5168	0.9951
7.0	0.4348	0.1467	0.9954	0.5085	0.3168	0.9882
DRMM						
4.0	0.2918	0.1169	0.9669	0.4955	0.5768	0.9953
6.0	0.1392	0.0919	0.7601	0.4765	1.3435	0.9986
7.0	0.1378	0.0756	0.6431	0.4650	1.3407	0.9982

Pseudo-first-order model: $\mathrm{Ln }\left({q}_{e}-q\right)=\mathrm{ln} \,{q}_{e}-kt$

Pseudo-second-order model: $\frac{t}{q} = \frac{1}{{k}_{2}{{q}_{e}}^{2}} + \frac{1}{{q}_{e}} t$

For pH 6.0 and 7.0 ± 0.2, with BC_500_, the *qe*_cal_ values with the Pseudo-first-order and Pseudo-second-order models were 0.4882, 0.4348, 0.5029 and 0.5085 mg g^−1^, with *R*^2^ values of 0.9877, 0.9954, 0.9951 and 0.9882, respectively (Table 4, Fig. 4a, b). The *k*_1_ values obtained from the Pseudo-first-order model were 0.2296 and 0.1467 min^−1^ for pH 6.0 and 7.0 ± 0.2, respectively. For the Pseudo-second-order model, the *k*_2_ values were 0.5168 and 0.3168 g mg^−1^ min^−1^ for pH 6.0 and 7.0 ± 0.2, respectively (Table 4, Fig. 4a, b).

For the DRMM, similar *qe*_cal_ concentrations were obtained for the three pH values when analysed with the pseudo-second-order model, with values of 0.4955, 0.4765, 0.4650 mg g^−1^ and *R*^2^ of 0.9953, 0.9986 and 0.9982, for pH 4.0, 6.0 and 7.0 ± 0.2, respectively. Additionally, the pseudo-second-order adsorption constants were 0.5768, 1.3435 and 1.3407 g mg^−1^ min^−1^ for pH 4.0, 6.0 and 7.0 ± 0.2, respectively (Table 4, Fig. 4c, d). The pseudo-first-order model reached only an *R*^2^ > 0.9500 for pH 4.0 ± 0.2 with a *qe*_cal_ value of 0.2918 mg g^−1^, and the *k*_1_ value was 0.1160 min^−1^ (Table 4). The *qe*_cal_ values for pH 6.0 and 7.0 ± 0.2 were < 0.2000 mg g^−1^, and the *R*^2^ were < 0.9000 (Table 4, Fig. 4c, 4d).

Since the adsorption of MG obtained with BC_300_ was the lowest of the three adsorbents evaluated, all results are in Table S1 and Figs S3a and S3b of Supplementary materials.

## Discussion

### Recuperación del lodo algal

#### Selection of coagulants for algal sludge recovery

In the recovery of microalgae from tertiary effluents to obtain higher value-added bio-products, must ensure that chemical additives or coagulants do not substantially alter the chemical composition of the algal biomass, the bio-products of interest and do not add new pollutants to the tertiary effluent that restrict its reuse (Gutiérrez et al. 2015; Lu et al. 2020; Lv et al. 2020). In the present investigation, the pH of effluent remains unadjusted, so no addition of acids or basics compounds occurs, and the initial pH of the effluent was 6.5 ± 0.8, allowing to recovery of > 90% of the algal biomass of *Chlorella* sp. when using 60 mg L^−1^ of the cationic coagulant. During the first 20 min of the process, the recovery percentage was 20% and increased with time, reaching 90% after 50 min (Fig. S1a Supplementary material). This percentage is within the required range for a cationic coagulant, according to König et al. (2014) and Nayak et al. (2019), who suggested that this type of product should recover approximately 90% of the biomass in less than 1 h (König et al. 2014; Nayak et al. 2019). Banerjee et al. (2014) used modified acacia gum to transform it into an organic cationic coagulant and able to recover 90% of the *Chlorella* sp. biomass after 30 min at pH 7.6, a value that did not correspond to the pH of the effluent from which they wanted to recover the *Chlorella* sp. cells (Banerjee et al. 2014).

The reasons why biomass recovery increased up to 90% were related to two mechanisms. First, the neutralisation of the negative-charge surface of the microalgae caused the repulsion between them to decrease, and the first aggregates began to form (König et al. 2014; Kadir et al. 2018; Mubarak et al. 2019). This mechanism depends on the concentration of the microalgae and is related to total solids (205 ± 10 mg L^−1^), settleable solids (146.3 ± 9.2 mL L^−1^), and the pH of the effluent (König et al. 2014; Nayak et al. 2019). The second mechanism is called bridging, in which the positively charged coagulant simultaneously binds to the surface of two or more microalgae and forms a bridge between them, increasing the size and weight of the polymer, allowing the aggregates to coalesce into larger flocs (Vandamme et al. 2013, 2014). When comparing the results of the present article with other published works, some of them have reported efficiencies higher than 90% by using lower coagulant concentrations. However, the pH of the aqueous solution or effluent must be modified by either acidifying or alkalinising with chemical compounds that could increase the toxicity of the effluent to treat (Gerchman et al. 2017; Yang et al. 2021a, b).

Ferric chloride allows higher than 90% biomass recovery but uses high concentrations of the inorganic compound (1000 mg L^−1^). A result that could be related to the fact that ferric chloride modifies the surface charge of the microalgae, decreasing electrostatic repulsion, but at strongly acidic or alkaline pH (Nayak et al. 2019); in the present study, pH 6.5 ± 0.2 was used (Fig. 1b). Even so, the results obtained in this work were similar to those obtained by Lal and Das (2016), that got a maximum efficiency of 98% in a culture of *Chlorella* sp. MJ 11/11, with 400 mg L^−1^ ferric chloride at pH 8.0 (Lal and Das 2016). On the other hand, ferric chloride doses of 1000 mg L^−1^ or higher could increase the concentration of dissolved solids, electrical conductivity and iron concentration in the effluent. Another negative aspect is that organo-metallic sludge, brown or brown effluent and acute toxicity in different animal and plant models may be generated (Lv et al. 2020; Saththasivam et al. 2022).

For the organic polymers evaluated, recovery percentages above 40% were unique for Guar gum and sodium alginate (Fig. 1c, d). Guar gum is a polysaccharide composed of galactose and mannose in a 2:1 ratio that forms gels with thickening and binding properties (Guzmán et al. 2013; Banerjee et al. 2014). Its coagulating capacity is due to the presence of positively charged carboxylated groups that exert an electrostatic attraction for the COO^−^ groups of galacturonic acid present in the cell wall of microalgae (Banerjee et al. 2014; Mokhtar et al. 2021). This interaction strongly depends on the pH-adjusted value achieved before the coagulation process. Therefore, in the present study, the recovery rate did not exceed 50% as the pH of the effluent was 6.5 ± 0.2.

On the other hand, alginate is an anionic polyelectrolyte negatively charged in a solution, capable of generating an electrostatic repulsion against *Chlorella* sp. cells. The *Chlorella* sp. cell wall contains sugar mixtures such as galacturonic acid, ribose, arabinose, xylose, glucose, galactose, and rhamnose (Weber et al. 2022). In particular, galacturonic acid has negatively charged COO– groups or residues, which are responsible for generating repulsion with the –COOH groups of alginic acid (Soto-Ramírez et al. 2021). Additionally, at pH below 7.0 ± 0.2, this polymer does not efficiently coagulate microalgae because acidic functional groups that interfere with electrostatic attraction predominate (Cancela et al. 2016).

### Characterisation of tertiary effluent before and after coagulation process

All parameters of the uncoagulated effluent were higher than when using the cationic coagulant to generate the coagulated effluent. It was related to the biomass presence of *Chlorella* sp. and to the intermediates of the carbon, nitrogen, phosphorus and sulphur cycles released by the algae or from the wastewater to treat in the tertiary reactor (Table 1). The decrease in COD (104.67 ± 3.67 mg L^−1^), TOC (5.33 ± 1.05 mg L^−1^) and NT (7.0 mg L^−1^) concentrations were directly related to the coagulation of *Chlorella* sp. cells since algal biomass composition is carbon, nitrogen and other organic compounds rich in phosphorus and sulphur (Lu et al. 2020). On the other hand, nutrients such as NO_3_^−^, NO_2_^−^, NH_4_^+^, SO_4_^−2^ and PO_4_^−3^ behaved as dissolved solids trapped in the aggregates (microalgae and the cationic coagulant); reason for which occurred a significant decrease of these nutrients, compared to the uncoagulated effluent (Chen et al. 2020). Mohseni et al. (2021) reported similar results; they used an organic coagulant (cationic starch) for *Chlorella* *vulgaris* and *Nannochloropsis* *salina* recovery from wastewater. The authors recovered more than 95% of the two microalgae, simultaneously removing the total phosphorus, nitrogen, and dissolved organic carbon. The high efficiency was probably due to the positive surface charge of the cationic starch, which favoured interaction with the microalgae, decreased the electrostatic repulsion, and that part of the nutrients finally was adsorbed to the coagulated microalgae (Mohseni et al. 2021).

### Biochar production and characterisation

The mixture of byproducts from secondary and tertiary sludge from wastewater treatment plants with lignocellulosic biomass from agro-industrial waste to produce biochar (by co-pyrolysis) is relevant for materials science and technology research (Peng et al. 2015; Arun et al. 2020; Vinod et al. 2020). These strategies allow for obtaining eco-friendly and sustainable bioproducts, easy to integrate into circular economy models (Leng et al. 2015; Soria-Verdugo et al. 2017; Bolognesi et al. 2021).

In the present study, lignocellulose (RM_1_/LCM) and pine bark (RM_3_/PB) meshes, mixed with algal sludge obtained by coagulation (RM_2_/CAS), were used (Table 2). The first two materials' contributions mainly involved lignin, cellulose, and hemicellulose (Castillo-Toro et al. 2021). These polymers have complex chemical structures, with stable chemical bonds (ether, carbon–carbon, β-glucosides, among others), high aromaticity (lignin), a high C:N ratios and low moisture percentages (9.04 ± 0.29 and 7.43 ± 0.57%, for RM_1_/LCM and RM_3_/PB, respectively) (Castillo-Toro et al. 2021; Chakraborty et al. 2021), (Table 2). In addition, the lignocellulose meshes (RM_1_/LCM) had dead fungal and bacterial biogenic biomass, which remained attached to the meshes after being used in wastewater treatment. These dead biogenic biomasses have chitin and peptide glucan-based polymers from the fungal and bacterial walls (Ardila-Leal et al. 2020; Soto-Ramírez et al. 2021); these are linked between subunits by β 1–4 glycosidic bonds (Dörr et al. 2019; Pathy et al. 2022). Although these bonds are less stable than those in lignin, they confer stability and rigidity to the fungi and bacteria cell walls while still alive (Dörr et al. 2019). Die microorganisms become biogenic biomass that provides potentially transformable carbon, representing an additional carbon source to improve the texture of the dried raw material converted into the two types of biochar (Ardila-Leal et al. 2020).

The microalgae coagulated sludge-based raw material (RM_2_/CAS) contributes less carbon than the other two raw materials (TOC: 2.72 ± 0.52%) (Table 2), which was related to the chemical composition of the microalgae as they can have variable carbon contents concerning nitrogen (Leng et al. 2015; Chen et al. 2018; Chakraborty et al. 2021; Pathy et al. 2022). Céspedes-Bernal et al. (2021) determined that *Chlorella* sp. sludge has a low percentage of carbon (6.9 ± 0.8%), and the ash content can vary but tends to be higher than in lignocellulosic biomass (Céspedes-Bernal et al. 2021). In microalgae sludge, the ash percentage oscillates between 6. 0 and 14%; this variation depends on the microalgae type, the coagulant type, and the use of the microalgae before the coagulation process, as they can accumulate inorganic nutrients that increase the ash content (Leng et al. 2015; Soria-Verdugo et al. 2017; Chen et al. 2018; Bolognesi et al. 2021; Chakraborty et al. 2021).

The mixture of RM_1_/LCM, RM_2_/CAS and RM_3_/PB allowed for obtaining a crude material with physical and chemical characteristics suitable for biochar production (DRMM) (Table 2). This mixture combines the chemical properties of structural polymers of plant origin (lignin, cellulose and hemicellulose) and structural polymers of microbial origin present in secondary and tertiary sludge (fungal, bacterial and algal wall). Because of this, TOC was higher (41.7 ± 0.77%) than in RM_1_/LCM and RM_2_/CAS, FC increased (24.5 ± 0.44%), VC decreased (65.4 ± 2%), and ash (10.1 ± 2.08) was similar to those obtained in RM_3_/PB (Table 2). Several authors have shown that raw materials obtained from microalgae, lignocellulosic biomasses and treatment plant sludge are promising feedstocks for biochar production. However, some of their physical and chemical properties can be improved when blended, as it decreases volatilisation losses; increases the fixed carbon content and biochar yields (Wang et al. 2016; Soria-Verdugo et al. 2017; Chen et al. 2018; Chakraborty et al. 2021).

Another production condition considered to improve the biochar quality was a pre-treatment, introducing it into an anaerobic hood to reduce the O_2_ before the DRMM co-pyrolysis process at 300 and 500 °C. These systems involve a chemical catalyst that generates hydrogen; this gas combines with O_2_ to form water and progressively reduces its concentration (Blanco-Vargas et al. 2022). Although it is difficult to eliminate all the O_2_ in an organic materials mixture, such as the one evaluated, it is possible to reduce it, allowing biochar production to be carried out under reduced oxygen conditions to decrease volatilisation losses and concentrate the solid products (Table 2). Additionally, production costs decrease as it would not be necessary to continuously introduce an inert gas into the muffle to maintain 100% oxygen-free conditions (Moreno-Bayona et al. 2019; Blanco-Vargas et al. 2022).

In both biochars, decrease the TOC (27.6 ± 0.68 and 31.4 ± 1.23%) percentages compared to DRMM (41.7 ± 0.77%), which could be related to the temperatures of biochar production. The thermal decomposition at 300 °C causes cellulose and hemicellulose depolymerization, generating changes at the volatile fraction (53.6 ± 0.18%) contrasting with the raw material volatile fraction (DRMM: 65.4 ± 2.07%) (Chen et al. 2018; Chakraborty et al. 2021). At 500 °C, cellulose, hemicellulose, and lignin undergo thermal decomposition.

However, due to the chemical characteristics of lignin (phenyl propane subunits linked by ether bonds), part of the aromatic rings reorganise and condense as part of the biochar (Blanco-Vargas et al. 2022). Being this the reason why the percentage of TOC was slightly higher than that obtained at 300 °C (31.4 ± 1.23%), and the VC percentage was low (51.2 ± 2.2%) when compared to the biochar produced at 300 °C (53.6 ± 0.18%), (Leng et al. 2015; Li et al. 2022).

In both biochars, increase the ash percentages (BC_300_: 30.6 ± 2.4 and BC_500_: 38.4 ± 2.3%) (Table 2). Sludge from secondary and tertiary wastewater treatment can have a high percentage of minerals and metals, as can wood by-products (although to a lesser extent); the mixture of these two materials increases the ash content, and the ash increases the pH and the electrical conductivity of the biochar (data not shown), (Leng et al. 2015; Bolognesi et al. 2021; Castillo-Toro et al. 2021; Céspedes-Bernal et al. 2021; Chakraborty et al. 2021). Concerning FC, the percentages were lower than those obtained by other authors who produced biochar with raw materials similar to the present study (Leng et al. 2015; Bolognesi et al. 2021; Chakraborty et al. 2021). The main reason for values ranging between 10.4 ± 1.1 and 15.8 ± 1.2% for BC_500_ and BC_300_ relates to the biochar production under reduced oxygen conditions but not in total anaerobiosis. The O_2_ presence inside the muffle favours the complete combustion up to CO_2_, and the FC percentage remains low. Another result related to the effect of biochar production under reduced oxygen conditions was the biochar yield, versus the percentage of biochar produced at 300 °C was 52.6 ± 3.5%, a higher value than that obtained at 500 °C (41.3 ± 2.4%), (Table 2).

### Evaluation of tertiary effluent and biochar for *Lolium* sp. seed germination

The two effluents (uncoagulated effluent and post-coagulated with the cationic coagulant at 60 mg L^−1^) favoured the germination of *Lolium* sp. seeds because they provided the necessary water, pH and low conductivity (Table 1); essential factors to facilitate moisture absorption, activate the embryo and carry out hydrolysis of the endosperm (Huang et al. 2019). As a result, the radicle emerged, elongated and formed the taproot within five days at 19 ± 2 °C. Subsequently, the root absorbed nutrients, such as nitrate and orthophosphates, present in the two effluents at concentrations that were not toxic to the seeds and the growing root (Tables 1, 3). According to resolution 1207 of 2014 (Colombian Ministry of Environment and Sustainable Development), the maximum permissible values of nitrates and nitrites for reusing effluent in agricultural irrigation should not exceed 5.0 mg L^−1^ (MADS 2014). On the other hand, the Environmental Protection Agency (EPA) of the United States has regulated that the maximum values of nitrates and nitrites in treated wastewater must not exceed 10 and 1.0 mg L^−1^, respectively (Bastian and Murray 2012). Based on the environmental regulations of Colombia and the United States, the coagulated effluent, free of *Chlorella* sp., could be reused in agricultural irrigation, generating a sustainable alternative for effluent reuse and preventing them from being discharged into sewage systems without further use.

The germination tests of *Lolium* sp, using DRMM and both biochars (BC_300_ and BC_500_), supplemented or not with beneficial bacteria, showed that DRMM did not favour the germination of *Lolium* sp. seeds (Table 3). In Fig. 2, d, purple, brown and red lignocellulose threads are observable in the DRMM; these colours suggest the presence of triphenylmethane and azo dyes, dye additives such as Lugol and Alcohol-Acetone. These dyes were toxic to both beneficial microorganisms and seeds of *Lolium* sp. (due to their chemical composition) (Tables 2, 3). Morales-Álvarez et al. (2016) reported that Crystal Violet and Malachite Green (MG) are toxic and affect the germination of *Lactuca* *sativa* seeds when used at 100, 50 and 25% (v/v) of dyes. Toxicity was related to the complex structure of triphenylmethane dyes based on the bonding between the central carbon atom and three aromatic rings (Morales-Álvarez et al. 2016). In 2022, the Environmental and Industrial Biotechnology Group (GBAI) of the *Pontificia* *Universidad* *Javeriana* (Bogotá, D.C., Colombia) used a mixture of lignocellulose meshes impregnated with synthetic dyes and coffee grounds as raw materials for biochar production. In that study, the raw material was allowed to germinate only 74% of the *Lolium* sp. seeds, and the germination was similar to the peat control, obtaining 95 and 97% germination for the biochar and peat, respectively (data not shown).

During the production of biochar at 300 and 500 °C, acid, aliphatic and volatile compounds (that could affect the germination of *Lolium* sp. seeds) were eliminated (Fig. 2e, f) and also part of the aromatic compounds from the adsorbed dyes and the lignin-rich biomass itself was concentrated and condensed in the two kinds of biochar (Fig. 2d–f); generating a porous material, with different particle size, larger surface area, rich in slow-release carbon, ash and water-holding capacity. These physical and chemical changes allowed high concentrations of beneficial bacteria to be immobilised (Table 2), maintaining their biological activity, such as phosphorus solubilisation, nitrogen fixation and the production of plant growth-promoting substances (Fig. 2g–i). Both bacterial cell walls and exopolysaccharides can have positive and negative surface charges, which interact with functional groups of the biochar and favour their adsorption, which depends on the pH of the aqueous suspension in which the bacteria were (pH 4.5 ± 0.2), the concentration of the bacteria, the concentration of biochar and the functional groups that the biochar has (Moreno-Bayona et al. 2019; Blanco-Vargas et al. 2022). Table 2 shows that two biochars had a pH below 7.0 (5.08 ± 0.08 and 6.78 ± 0.01, for BC_300_ and BC_500_, respectively), and the suspension of microorganisms had a pH of 4.5 ± 0.2. Under these conditions, the biochar possibly acquired more positively charged functional groups (amino, carboxyl, phosphate and sulphate groups), which interacted with the negative charges of the bacterial wall, favouring adsorption without losing biological activity (Blanco-Vargas et al. 2022).

On the other hand, both biochar alone and co-inoculated with beneficial bacteria favoured the germination of *Lolium* sp. seeds, obtaining higher percentages than the soil control (90 ± 2.0). The use of two types of biochar could generate a synergistic effect with the bacteria, in which the biochar acted as solid porous substrates that allowed air diffusion and moisture retention. Additionally, biochar had high percentages of ash (Table 2), in which macronutrients such as N, P and K and micronutrients such as Zn, Mn, Fe, Bo, and Cu could be present. These elements could be more available to the germinated seeds due to the biological activity of the microorganisms co-inoculated in the biochar. Bacteria of the genera Pseudomonas and Bacillus are phosphorus solubilisers due to the production of readily dissociable organic acids, the release of protons (H^+^) and the production of chelating compounds (Corrales et al. 2014; Restrepo-Franco et al. 2015). As a result of these mechanisms, the inorganic forms of phosphorus in the ashes probably were solubilised in both biochars and orthophosphate ions (the assimilable form of phosphorus for plants) were released (Moreno-Bayona et al. 2019; Blanco-Vargas et al. 2022). On the other hand, *Pseudomonas* *fluorescens* produces plant growth-promoting substances such as gibberellins; these phytohormones are crucial for germination and root growth, participating as secondary metabolites in several processes related to plant resistance to different environmental factors (Zhou et al. 2016). *Azotobacter* sp. has two mechanisms to promote germination and initial root growth of *Lolium* sp.; the first concern its ability to produce phytohormones, and the second is to fix atmospheric nitrogen without the need for a symbiotic relationship with plants (Hindersah et al. 2020; Sumbul et al. 2020).

### Evaluation of biochar and DRMM as adsorbents

Under the conditions evaluated in the present study, there are two aspects to consider; the first related to the raw materials used, which were a mixture of biogenic biomasses and lignocellulosic biomasses; therefore, the functional groups present in the DRMM and biochar may vary, and the adsorption of MG is not because of a single material of those used. Second, the adsorption of MG onto an adsorbent (DRMM, BC_300_ and BC_500_) depends on the functional groups present on the adsorbent surface and the ionisation state of the dye (Chen et al. 2018). In the evaluation of DRMM and biochar at pH of 4.0, 5.0, and 7.0 ± 0.2, MG was in its ionised form, which makes it behave as a cationic or positively charged dye because the pH of the three aqueous solutions was lower than the pK of MG (6.9 for pKa1 and 10.0 for pKa2), (Rubio-Clemente et al. 2021). The MG positively charged can interact with the negatively charged surface of DRMM and biochar through electrostatic interactions (Chen et al. 2018).

In Fig. 3a, b, both DRMM and BC_500_, had higher adsorption capacity at the three pH values evaluated, possibly in DRMM negatively charged functional groups such as –OH, –COOH and –NH_2_ were present, which by electrostatic attraction bound with MG (Zhang et al. 2017). These groups come from the biogenic biomass of *Chlorella* sp. (RM_2_/CAS) and biogenic fungal/bacterial biomass on lignocellulose meshes (RM_1_/LCM). Yang et al. (2021b) produced and characterised different types of biochar at 200, 350 and 550 °C, using *Chlorella* sp. and *Spirulina* sp. biomass. The authors determined the presence of these functional groups in the two microalgae without heat treatment (raw materials) and by evaluating the adsorption of methylene blue (cationic dye), suggesting that these groups could participate in dye removal (Yang et al. 2021b). Additionally, Pathy et al. (2022) used biochar and a composite from a consortium of microalgae (*Chlorella* sp., *Scenedesmus* sp., *Synechocystis* sp., and *Spirulina* sp.) and a mixture of bacteria and yeasts used for the production of fermented Kombucha (SCOBY), for the adsorption of MG. The authors detected the presence of different functional groups in the raw materials associated with the microalgae (S=O, C=C, OH, among others), and in SCOBY, they found a large O–H stretching. These functional groups decreased when producing the different types of biochar; however, the authors suggest that oxygen-containing groups, aromatic groups, phenolics and carboxyl groups on the outside of the biochar could play a crucial role in the removal of contaminants through adsorption (Pathy et al. 2022).

On the other hand, lignocellulosic biomass (RM_1_/LCM and RM_3_/PB), rich in cellulose, hemicellulose and lignin, could also have negatively charged hydroxyl groups (–OH) that participated in the adsorption of MG. Rubio-Clemente et al. (2022) produced two types of biochar from *Pinus* *patula* wood pellets and chips. In the characterisation by infrared spectroscopy, they determined that –OH groups and –OH stretching were present in raw and unheated materials (Rubio-Clemente et al. 2021).

BC_500_ also removed MG at all three pH values evaluated; the result suggests that the biochar had negative surface charges, possibly associated with hydroxyl (–OH) and carboxyl (–COOH) groups, from the Caribbean pine bark used as one of the raw materials (Céspedes-Bernal et al. 2021), (Fig. 3b).

Ahmad et al. (2021) produced an activated carbon from *Hevea* *brasiliensis* roots, which have cellulose, hemicellulose and part of lignin. The authors observed the presence of carboxyl-carbonate structures in both the raw material and the biochar but in smaller proportions. These compounds with carboxyl groups may act as additional negatively charged functional groups for the adsorption of cationic dyes (Ahmad et al. 2021). Another feature that could favour the adsorption of MG was the presence of ashes in a higher proportion than DRMM and BC_500_ (Table 2). Ashes are a mixture of mineral compounds concentrated in biochar; these compounds with a positive or negative charge are a fraction of the biochar with adsorption capacity (Chen et al. 2018). Finally, BC_500_ had a smaller particle size, which means an increase in porosity and surface area, complementing the adsorption capacity of BC_500_.

When analysing the results of the pseudo-first-order and pseudo-second-order models at the three pH values evaluated (Fig. 4, Table 4), the results suggest that the adsorption of MG onto DRMM and BC_500_ could occur in two ways, physical adsorption (physisorption) and chemical adsorption (chemisorption), which could be complementary to each other and varied according to the adsorbent. Physisorption was due to Van der Waals forces, and chemisorption involved bonds from the MG and the functional groups on the adsorbent surface (Pathy et al. 2022).

For BC_500_ (best adsorbent), the MG removal could occur through both mechanisms since the *R*^2^ values were higher than 0.9300 for the two models evaluated (Table 4). With the Pseudo-first-order model could be assumed that the rate of MG adsorption was proportional to the availability of active sites on BC_500_ being pH 4.0 ± 0.2, which favoured the highest availability of active sites and thus generating the highest value of *qe*_cal_ (0.5773 mg g^−1^), (Table 4, Fig. 4a). Concerning the Pseudo-second-order model, a complementary chemisorption process between the dye and the biochar could occur at the three pH values evaluated until reaching the equilibrium state (Table 4, Fig. 4b).

The combination of physical and chemical adsorption mechanisms was also for the DRMM at pH 4.0 ± 0.2. Since *R*^2^ values for the Pseudo-first-order and Pseudo-second-order models of 0.9669 and 0.9953, respectively, were obtained (Table 4, Fig. 4c, d). At pH 6.0 and 7.0 ± 0.2, the chemosorption process possibly predominated, because the *R*^2^ values for the pseudo-second-order model were 0.9986 and 0.9982, respectively (Table 4, Fig. 4c, d).

Despite the results obtained with the Pseudo-first-order and Pseudo-second-order models, which suggest a possible combination between physisorption and chemosorption, from MG to DRMM and BC_500_, it could not stay secured that the two adsorbents behaved as a homogeneous monolayer or a heterogeneous multilayer. For this, it would be necessary to perform equilibrium adsorption isotherms by applying the Langmuir and Freundlich models. Leng et al. (2015) produced biochar similar to the one evaluated in this study; the authors used microalgae, lignocellulosic biomass and sludge from wastewater treatment plants to generate different types of biochar. Subsequently, they used them as adsorbents for the removal of the cationic dye methylene blue and also to determine that this dye best fitted the Langmuir model and suggested the adsorption process explanation through a chemosorption mechanism associated with a homogeneous monolayer, in which ion exchange reactions involves hydrogen bridge formation and electrostatic interactions to adsorb the dye (Leng et al. 2015).

## Conclusions

In this study, the thermal conversion of semi-solid sludge generated in a pilot plant for the treatment of non-domestic wastewater was successful, demonstrating that the post-coagulation effluent may be for the seedling growing substrate of rhizomatous herbs of the Poaceae family. Due to these results, eco-friendlier bio-products were obtained, with higher added value that can integrate into circular economy models. The effluent derived from the coagulation of *Chlorella* sp. had physical and chemical characteristics that favoured the germination of *Lolium* sp. seeds, generating alternative reuse for this effluent, thus preventing it from being discharged into sewage systems. Concerning the biochar produced by co-pyrolysis, two novel biomaterials allowed the germination of *Lolium* sp. seeds. Both biochars acted as suitable organic/mineral supports to immobilise or co-inoculate beneficial microorganisms for plants and soil, acting as multipurpose materials for peat replacement, an organic substrate overexploited worldwide in the propagation of plant material. Finally, two kinds of adsorbents obtained favoured the removal of a cationic dye of high environmental impact, such as malachite green.

### Supplementary Information

Below is the link to the electronic supplementary material.

Supplementary file1 (DOCX 989 KB)
