# Supplementary material for: Use of biochar and a post-coagulation effluent as an adsorbent of malachite green, beneficial bacteria carrier, and seedling substrate for plants belonging to the poaceae family
Source: 3 Biotech. 2023 Nov 3;13(12):386. doi: 10.1007/s13205-023-03766-x (PMC10624780; doi:10.1007/s13205-023-03766-x)
Supplement: Supplementary file 1 — Supplementary file1 (DOCX 989 KB) [file 13205_2023_3766_MOESM1_ESM.docx]

**Supplementary Material**

**Fig. S1 The percentage of biomass recovery as a function of time**

The recovery efficiency curve was performed over time, consisting of fast mixing (200 rpm) for the first minute and then slow mixing (100 rpm) until the 60th minute. The measurements at minutes 90 and 120 corresponded to static sedimentation done for 1 hour. Fig. S1a shows that the recovery efficiency increased with time, exceeding 90 % efficiency after one hour of mixing and reaching its highest value (97 %) during static sedimentation at minute 120. Figs. S1b and S1c, show the compact flocs formed after 120 minutes.


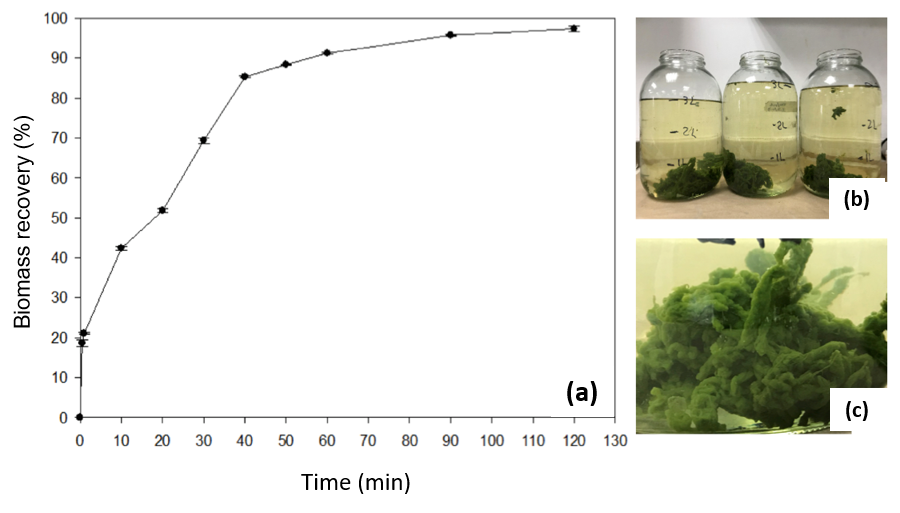


**Fig. S2 Microscopic appearance**

Microscopic appearance (40X) of *Chlorella* sp. cells, without coagulant addition (Fig. S2a). Addition of a cationic coagulant at a concentration of 60 mg L^-1^, pH 6.5 ± 0.2 (Fig. S2b). Addition of FeCl_3_-6H_2_O at a concentration of 1000 mg L^-1^ at pH 6.5 (Fig. S2c).


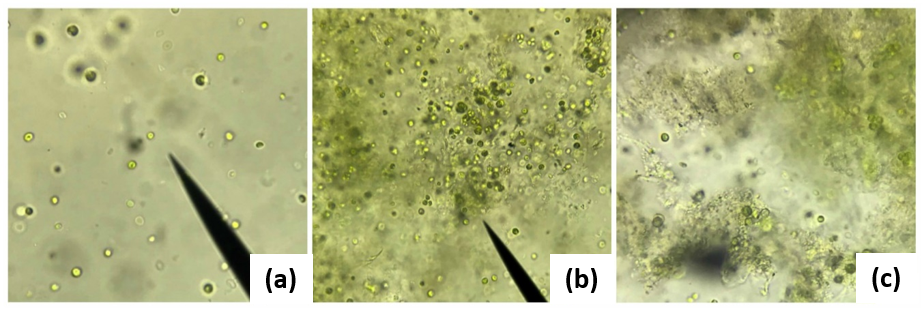


.

**Fig. S3 The adsorption process from MG to BC_300_**

BC_300_ adsorbed the highest amount of MG at pH 7.0 ± 0.2, with a *qe* _cal_ value of 0.4323 mg g^-^1, R^2^ of 0.9542, and a Pseudo-first-order constant of 0.0293 min^-1^. Continuing with the Pseudo-first-order model for pH 4.0 and 6.0 ± 0.2, the *qe* _cal_ values were 0.3146 mg g^-1^ (R^2^ of 0.9736) and 0.3402 mg g^-^1 (R^2^ of 0.9683). The constants values were 0.0374 and 0.0334 min^-1^ (Supplementary Table S1, Fig. S3). With the pseudo-second-order model, only an R^2^ higher than 0.9500 resulted in pH 4.0 ± 0.2, with a *qe* _cal_ value of 0.1772 mg g^-1^ and a *k_2_* of 0.1395 (g mg^-1^ min^-1^), (Supplementary Table S1, Fig. S3).

BC_300_ possibly had higher positive surface charges than negative ones, determining the electrostatic repulsion generated between the biochar and the cationic dye (Fig. S3). In addition, biochar produced at 300 °C had low ash content (Table S1). The combination of these characteristics could be the reason for the slow adsorption than those obtained with DRMM and BC_500_.

**
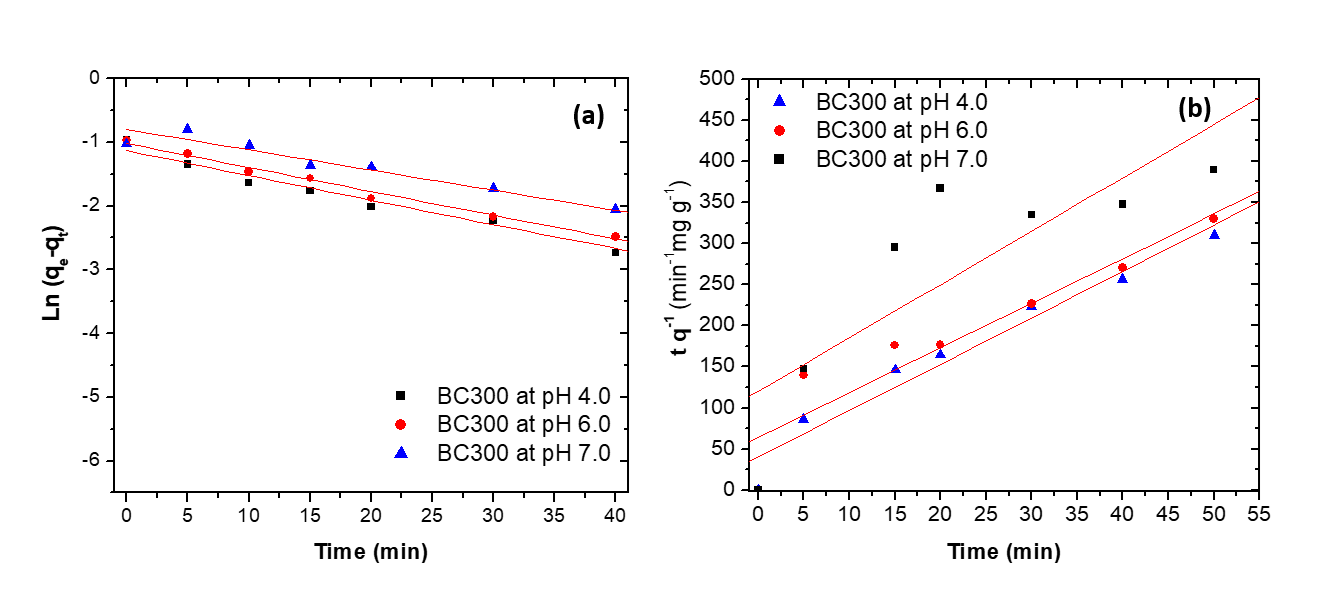
**

**Fig. S3** Kinect models and rate constants. Pseudo-first-order models for BC_300_ (**a**), Pseudo-second-order models for BC_300_ (**b**). at pH of 4.0, 6.0 and 7.0 ± 0.2

**Table S1.** Kinetic model and rates constants of the Pseudo-first-order and Pseudo-second-order models for BC_300_ at pH of 4.0, 6.0 and 7.0 ± 0.2

| pH ± 0.2 | Pseudo-first-order | | | Pseudo-second-order | | |
| --- | --- | --- | --- | --- | --- | --- |
|  | *q_e_*  mg g^-1^ | *k_1_*  min^-1^ | R^2^ | *q_e_*  mg g^-1^ | *k_2_*  g mg ^-1^ min^-1^ | R^2^ |
| BC_300_ | | | | | | |
| 4.0 | 0.3146 | 0.0374 | 0.9736 | 0.1772 | 0.1395 | 0.9557 |
| 6.0 | 0.3402 | 0.0334 | 0.9683 | 0.1841 | 0.0842 | 0.8858 |
| 7.0 | 0.4323 | 0.0293 | 0.9542 | 0.1542 | 0.0537 | 0.6839 |

_Pseudo-first-order model:_ $Ln \left( qe-q \right)=\ln qe-kt$

_Pseudo-second-order model:_ $\frac{t}{q} = \frac{1}{k_{2}{qe}^{2}} + \frac{1}{qe} t$
